# Proteome-Based Biomarkers for Alzheimer’s Disease: Old Acquisitions and Innovative Proposals

**DOI:** 10.3390/ijms262311654

**Published:** 2025-12-01

**Authors:** Valeria Magnelli, Corinna Anais Pagano, Maurizio Sabbatini

**Affiliations:** DiSIT—Dipartimento di Scienze e Innovazione Tecnologica, University of Piemonte Orientale, Viale Teresa Michel 11, 15121 Alessandria, Italy; corinnaanais.pagano@uniupo.it (C.A.P.); maurizio.sabbatini@uniupo.it (M.S.)

**Keywords:** Alzheimer’s disease, biomarker, proteomics, cerebrospinal fluid, A*β* protein, neurofibrillary tangles, plasma

## Abstract

Alzheimer’s disease (AD) is one of the most widespread neurodegenerative disorders, primarily affecting individuals over the age of 65. It is characterized by severe cognitive impairment, memory loss, difficulties in performing daily activities, ventricular enlargement, and ultimately, dementia. AD is associated with the accumulation of amyloid β(Aβ) protein plaques, intracellular neurofibrillary tangles (NFTs), progressive inflammation, and impairment of both synaptic transmission and mitochondrial function. Due to the limited diagnostic tools available for detecting the disease in its early stages, proteomic biomarkers have gained paramount importance, as they can monitor prodromal molecular alterations linked to AD. Furthermore, proteomic biomarkers can facilitate the longitudinal assessment of disease progression and contribute to the development of personalized therapeutic strategies before the devastating onset of dementia. Research has primarily focused on identifying proteomic biomarkers in cerebrospinal fluid (CSF) and plasma, as discussed in this review, but also in alternative matrices such as saliva and urine. These studies highlight both the high potential of proteomic approaches and the ongoing challenge of identifying clinically available, sensitive, and specific biomarkers for the various stages of the disease.

## 1. Introduction

Alzheimer’s disease (AD) is the fastest-growing neurological degenerative condition affecting a large number of people worldwide each year. AD is the most common form of dementia, accounting for about 60% of all clinically diagnosed cases, and typically develops after the age of 65. It is a severely disabling condition characterized by brain changes that begin years or even decades before clinical symptoms such as memory loss and confusion appear [1]. The disease involves widespread neuronal cell death, leading to cognitive impairment, memory loss, and a decline in daily abilities [2,3]. Once AD is diagnosed, it follows an irreversible course, resulting in extensive brain damage and a marked reduction of gray matter in the hippocampus and entorhinal cortex, regions crucial for long-term memory circuits [4].

### 1.1. AD Overview: Aβ Cascade and Clinical Signs

AD is a complex multifactorial disorder characterized by the involvement of multiple factors in its onset. Clinically, AD manifests as an early impairment of short-term memory, which gradually evolves over several years into profound deficits in episodic and semantic memory, executive function, language, and eventually severe behavioral and neuropsychiatric disturbances [1,3,5].

Among the earliest biological alterations, amyloid-β (Aβ) accumulation and tau hyperphosphorylation (p-tau) remain central hallmarks of AD pathology, although they are now considered part of a broader, multifactorial network of disease processes [6].

The accumulation of Aβ species—particularly Aβ42—can begin years before clinical symptoms and contributes to synaptic dysfunction and progressive neurodegeneration [7,8,9,10,11]. Microglial activation initially counteracts plaque deposition but later exacerbates tau pathology and neuronal injury [12,13,14,15]. Progressive propagation of Aβ and tau aggregates across brain regions leads to synaptic failure, neuronal loss, and gradual cognitive decline. Additional pathological mechanisms include calcium dyshomeostasis, neuroinflammation, mitochondrial dysfunction, and oxidative stress [16,17,18,19,20,21]. As disease progression continues, structural changes become evident, with early involvement of the enthorinal cortex and hippocampus followed by widespread cortical atrophy, ventricular enlargement, and functional network disruption [22,23,24]. Structural and functional neuroimaging (magnetic resonance imaging, MRI; positron emission tomography, PET) as well as fluid biomarkers have become essential tools for early diagnosis and stratification [25,26].

Genetic factors also modulate disease risk: the apolipoprotein E (ApoE) ϵ4 allele is the strongest known genetic susceptibility factor for late-onset AD [27,28], whereas mutations in amyloid precursor protein (APP), presenilin-1 (PSEN1), and presenilin-2 (PSEN2) cause rare familial forms of early-onset AD [29].

From a clinical perspective, diagnostic criteria have evolved toward biomarker-based frameworks, such as those proposed by the National Institute of Aging–Alzheimer’s Association (NIA-AA) and the International Working Group, which integrate cognitive evaluation with imaging and fluid biomarkers to identify AD across its continuum from preclinical phases to dementia [30]. This modern, multi-modal approach aligns with recent therapeutic strategies targeting early pathological events, reinforcing the need for timely diagnosis in order to maximize the potential benefits of emerging disease-modifying treatments [31].

### 1.2. Recent Therapeutical Approaches in AD Handling

AD is a condition with a significant social and welfare impact that seriously compromises morphological and functional aspects of the central nervous system (CNS). Thus, it is a paramount request to find out and set new therapies to limit the diffusion of this widespread form of dementia. Scientists have been seeking treatment for decades. Since Aβ plaques accumulate and expand long before symptoms appear, most efforts aimed to clear them from the brain and prevent the formation of new ones. Aducanumab was a human monoclonal antibody that specifically targeted Aβ plaques. It has been approved in 2021, but its high costs and the reported adverse events raised questions about its safety and cost-effectiveness. Moreover, there was little evidence that Aβ clearance correlated with slowed cognitive or functional decline. A systematic review is reported in Rahman et al. (2023) [32]. Different projects have been started to set new therapeutical drugs, and a recent pipeline is reported in Cummings et al. (2025) [33]. One of the outstanding types of research is the Clarity AD project, a randomized, 18-month, multi-center, double-blind study that appears to show positive results for the efficacy of the experimental drug Lecanemab, an anti-amyloid antibody. It involved 1795 patients with mild cognitive impairment due to AD or mild AD, with positive amyloid biomarkers. The results indicate that Lecanemab provides benefits in different aspects ranging from cognitive function to disease progression and biomarkers. It helps in the relative preservation of the quality-of-life offering improvements to patients, care partners, and society [34].

## 2. AD Biomarkers

It is crucial to identify valid molecular tools in screening programs to detect individuals at the early stages of AD, long before the appearance of clinical symptoms, in order to diagnose the disease unambiguously and assess its potential progression rate. Biomarkers serve as indicators of pathological states and can now be reliably measured in patient fluid samples to suggest the presence or absence of the disease or the likelihood of developing AD later. These biomarkers help to track the progression of the disease from presymptomatic stages to mild cognitive impairment (MCI) and to more advanced stages, including AD dementia. At the same time, a validated biomarker should distinguish AD dementia from other types of dementia, such as frontotemporal lobar degeneration (FTLD) or Lewy body dementia (LBD) [35]. Furthermore, the discovery of molecular targets could facilitate the implementation of appropriate medical interventions before the brain degeneration characteristic of AD becomes irreversible, supporting the evaluation of patient condition.

### 2.1. Categories of Proteomic Biomarkers

Proteins are highly sensitive in diagnosing specific diseases and, for this reason, proteomics represents the most promising approach to identify AD biomarkers. AD proteomic biomarkers include proteins derived from brain homogenate [36], cerebrospinal fluid (CSF) [37], blood [38] and even saliva [39] and urine [40].

Proteomic biomarkers can be classified into three categories: prognostic, diagnostic, and treatment-predictive, according to the type of information they provide [41]. A prognostic biomarker provides information about the likely course of a disease in an untreated individual, regardless of the therapy applied. In other words, it helps identify patients at higher or lower risk of disease progression, recurrence, or overall outcome. For example, certain protein expression patterns may indicate whether a lesion is more likely to progress aggressively or remain stable. A diagnostic biomarker aids in detecting or confirming the presence of a disease or pathological conditions. In the context of proteomics, these biomarkers are crucial for distinguishing diseased tissue from healthy tissue with higher specificity and sensitivity than conventional methods. They can also facilitate early disease detection, even when clinical signs are subtle or absent, thereby improving the chances of effective intervention. A treatment-predictive biomarker (sometimes referred to as a predictive biomarker) provides insight into the likelihood of a patient responding to a particular therapeutic intervention. Such biomarkers can guide clinicians in selecting the most appropriate treatment, reducing unnecessary exposure to ineffective therapies, and personalizing care. For instance, the presence or absence of specific protein signatures may predict whether a patient will benefit from regenerative approaches, surgical interventions, or targeted pharmacological therapies. Together, these three categories of proteomics biomarkers represent an important milestone in the advancement of personalized and precision medicine [42].

### 2.2. Proteomics in Personalized and Precision Medicine

Proteomics enables a better definition of the final product of a gene, its role in protein networks, and its qualitative and quantitative changes following pathological processes. Proteomic biomarkers have significantly advanced the in vivo diagnosis of AD by uncovering the major proteins involved in disease pathogenesis. The important developments in mass spectrometry (MS), along with improved analytical techniques and dedicated software, have provided deep insights into AD mechanisms. Bioinformatics and data analysis, now strengthened by deep learning and artificial intelligence (AI), are key components of AD proteomics [43,44]. One major advantage is that proteomics allows the quantification of thousands of proteins and the detection of post-translational modifications, such as phosphorylation, methylation, and oxidation, which are known to play pivotal roles in AD, providing crucial insights into the molecular mechanisms driving AD pathogenesis [45]. The implementation of proteomics techniques and bioinformatic tools in biomarker research has gained widespread acceptance because it enables large-scale detection and identification of proteins [46], thereby increasing the likelihood of discovering novel biomarkers [47]. Proteomics studies benefit from multiple approaches, ranging from two-dimensional electrophoresis (2DE) and MS to protein microarrays [42].

In 2014, the National Institutes on Aging initiated a multi-approach program aimed at discovering new biomarkers [48]. The current status of this program relies on the capability for high-throughput quantification and comparison of thousands of proteins from samples of healthy and AD individuals. This review will focus on the proteomics biomarker landscape for AD in CSF and blood, considering some of the most extensively investigated pathological pathways that characterize this widespread disease.

## 3. Proteomics for Alzheimer’s Disease Research

Proteomic techniques have been applied to search for AD biomarkers in different body compartments, with CSF and blood being the most extensively investigated. CSF is the optimal sample, as it flows directly through the CNS, where AD primarily develops, and contains higher concentrations of neurodegenerative biomarkers. The main limitation of its use is that sample collection via lumbar puncture is relatively invasive; for this reason, blood is often preferred. Blood is more easily accessible and CNS biomarkers can be detected due to alterations of the blood–brain barrier (BBB) [49], making it suitable for AD proteomics analysis as well. When the disease begins, many proteins alter their expression profiles, revealing fundamental differences between healthy individuals and AD patients [50]. One of the more accessible approaches for the identification of AD potential biomarkers is 2DE-based proteomics combined with MS analysis, which offers high sensitivity and allows for the identification of thousands of proteins [51]. There are two primary approaches to identifying and characterizing proteins obtained from cells, tissues, or biofluids. The first is a bottom-up approach, which involves proteolytic digestion of the extracted proteins before MS analysis. Its main limitation is the low sequence coverage of the original protein, which can result in the loss of significant information. The second is a top-down workflow, a method well-suited for identifying and characterizing intact proteins from complex biological mixtures, including their post-translational modifications, as it provides access to the complete protein sequence [52]. A major challenge in protein biomarker research is the detection of low-abundance proteins, which can be masked by high-abundance proteins. For this reason, recent technological advancements focus on sample enrichment to better detect proteins of interest [53]. The availability of human protein databases facilitated more comprehensive studies of the AD proteome and its sub-proteomes of specific regions of interest. The sub-proteome of amyloid plaques, referred to as amyloidome, revealed approximately 900 proteins differentially expressed in rapidly progressive versus sporadic AD [54].

Brain-derived samples from AD and control subjects with known ApoE gene status allowed the identification of over 5000 proteins in the synaptic sub-proteome. Proteins involved in synaptic activity, such as neurexin 2 and neurogranin, resulted in downregulation, and proteins involved in the immune system and neuroimmune signaling were dysregulated [55]. MS techniques have enabled the identification of thousands of proteins involved in AD, contributing to multiple molecular processes, including synaptic activity [56,57], mitochondrial function [58], metabolism [59], and glial biology [60]. The ability to detect proteins with different expression levels between AD patients and healthy individuals, starting from the earliest pathological changes, represents a promising avenue for AD diagnosis and prognosis.

## 4. Cerebrospinal Fluid (CSF) Biomarkers

MS-based proteomics of CSF enables the analysis of thousands of proteins, thereby increasing the potential to identify novel disease biomarkers and allowing for selective, precise quantification that provides both protein identity and abundance [61,62,63]. Over time, MS-based research has yielded multiple promising results, and several potential biomarkers have been proposed. However, it is important to emphasize that a substantial portion of these findings comes from experimental studies or small cohorts and requires validation in larger clinical populations before it can be considered ready for diagnostic use.

### 4.1. Classical Biomarkers in CSF of AD Patients

The protein changes that characterize the neurodegenerative process of AD are best observed in CSF, which is the most reliable biofluid for identifying AD proteomic biomarkers, as it directly reflects the main neuropathological processes occurring in the CNS [64]. In the amyloidogenic cascade, APP and secretase enzymes generate two main fragment isoforms: the soluble Aβ40 and the insoluble, hydrophobic Aβ42. Aβ42 aggregates more readily, leading to its reduced concentration in CSF, likely due to sequestration within amyloid plaques. Decreased concentrations of CSF Aβ42 to approximately 50% have been reported in patients with AD with respect to healthy controls [65]. Different clinical studies have reported significant decrease over the years in Aβ42 levels, which are related to disease progression from MCI to AD [66,67]. Nonetheless, these findings derive from heterogeneous methodological settings, and variability in assay techniques may affect reproducibility, requiring careful standardization before widespread clinical implementation.

However, Aβ42 reduction is not specific to AD, as it can also occur in other CNS pathologies such as tuberculous meningitis [68] and Parkinson’s disease [69]. Aβ40 peptide is not as pathogenic as Aβ42 in developing amyloid pathology [70], and its selective increase may actually reduce the risk of AD development [71]. Aβ42/Aβ40 ratio has been proposed as a more accurate CSF biomarker for AD than Aβ42 alone [72]. It offers greater accuracy for the identification of amyloid plaque in early AD and better discrimination between AD and non-AD dementias [73,74], especially at the early stages of the disease [75]. Despite its strong performance, widespread adoption still depends on inter-laboratory harmonization of assays and further validation across diverse cohorts.

Soluble and insoluble Aβ fragments isolated from plaques were first identified using liquid chromatography (LC)–electrospray ionization (ESI)–mass spectrometry (MS) [76] and matrix-assisted laser desorption ionization (MALDI)–time-of-flight (TOF)–MS [77]. Before 1992, these were the only well-characterized AD-associated proteins, owing to the limited availability of protein databases. The completion of the Human Genome Project in 2003 provided comprehensive protein databases, laying the groundwork for full-scale proteomic analysis. Recently, Aβ peptides have been accurately quantified with different techniques [78,79].

The Aβ fragments derive at first by the action of the beta secretase 1 (BACE1) enzyme, which has been shown to have stronger activity in AD patients with respect to healthy people [80]. More recently, BACE1 activity has been associated with increased AD development risk [81], but current evidence remains inconsistent and limited by methodological heterogeneity: thus, BACE1 can not yet be considered a clinically actionable biomarker.

Total tau proteins (t-tau) promote microtubule stabilization, but become dysfunctional when phosphorylated (p-tau) [82]. t-tau levels increase in AD [83], likely reflecting neuronal loss, and are associated with altered CSF proteins plasticity and BBB dysfunction [84]. p-tau proteins are associated with pretangles formation [85] and have been investigated by MS [86]. However, CSF t-tau and p-tau correlate only weakly with NFTs’ pathology [87]. Isoforms p-tau181, p-tau231, and p-tau217 are all elevated in AD patients, with p-tau181 now used in routine biochemical assessments [88,89]. Although these proteins are the most validated CSF biomarkers to date, they still require careful interpretation, as pre-analytical differences and assay-specific variability may impact diagnostic accuracy in real-world settings.

Currently, Aβ, t-tau, and p-tau represent the gold-standard biomarkers for AD diagnosis [51,90] and for predicting progression from MCI to AD [91]. Their combined profile (low Aβ42 with high t-tau/p-tau) represents a robust biomarker profile for AD-related neuropathology able to distinguish AD with high sensitivity [92]. CSF analyses show concordance between this biomarker profile and amyloid burden in cortical biopsies [93].

ApoE4 is the strongest genetic risk factor for AD and is associated with amyloid pathology at any cognitive stage [94]. ApoE4 undergoes proteolytic cleavage, generating fragments that affect mitochondria and intracellular proteins [95,96], with region-specific patterns [97]. ApoE4 may also act as a transcription factor [98]. CSF ApoE correlates with AD biomarkers, but despite these associations, ApoE levels lack diagnostic specificity and do not reflect neuropathological burden, limiting its translational value [99].

Heparan sulfate proteoglycans (HSPGs) play a significant role in amyloid plaque formation [100,101], impairing the clearance pathways [102]. In particular, syndecans, membrane-bound HSPGs, interact with Aβ through their heparan sulfate (HS) chains, promoting Aβ oligomerization and fibril formation. Furthermore, syndecans regulate the activation of microglia and astrocytes, influencing the inflammation process [103]. Although these findings highlight interesting mechanistic insights, studies are predominantly preclinical or observational, and their use as clinical biomarkers remains speculative.

### 4.2. Other Biomarkers in CSF of AD Patients

Although Aβ and tau proteins remain the most established CSF biomarkers, they capture only a part of the biological complexity of AD. Many proteins have been identified within plaques [54]. Several studies have explored inflammatory and oxidative biomarkers as parallel contributors to AD onset.

Neuroinflammation plays a key role in AD [104], with microgliosis and astrocyte activation contributing to neuronal damage [105]. Variants of the receptor expressed on myeloid cells (TREM2) increase AD risk [106,107]. The soluble form of TREM2 correlates with Aβ42 and tau, but results are inconsistent across studies, partly due to differences in assay sensitivity, cohort composition, and disease staging [108,109].

Chitinase-3-like-protein-1 (YKL-40) reflects astrocyte activation [110] and correlates with tau from early disease stages [111], making it a promising biomarker for AD progression [112]. Neverthless, YKL-40 elevation is is also observed in multiple neuroinflammatory conditions, limiting its diagnostic specificity.

Osteopontin is elevated in early AD and progressive cognitive decline [113], but evidence remains based on relatively small and heterogeneous cohorts, and its clinical applicability must be interpreted with caution.

Inflammatory proteins, interleukin-10 (IL-10) [114], macrophage migration inhibitory factor (MIF) [115], and monocyte chemoattractant protein-1 (MCP-1) [116], differ between AD and non-AD patients. However, these proteins require further investigation before they can be considered reliable biomarkers. Overall, although several inflammatory markers seem promising, many remain in exploratory phases and their translation to clinical use will require standardized methodologies, large-scale validation, and longitudinal studies (Table 1).

Oxidative stress biomarkers have also been proposed [117,118]. Malondialdehyde is elevated in MCI, especially in younger subjects [119], but oxidative markers lack disease specificity, as oxidative damage is shared across many neurodegenerative conditions.

Neurofilament light chain (NfL) reflects axonal degeneration and increases in AD and other disorders [120]. Although NfL is not diagnostic for AD, it is a useful monitoring tool and the NfL/Aβ42 ratio predicts atrophy and cognitive decline [121]. Still, its limited specificity means Nfl should be integrated with other biomarkers and imaging findings rather than be used alone in clinical decision-making.

## 5. Plasma Biomarkers

AD is characterized by damage to the BBB, leading to increased permeability of the vascular endothelium [122,123,124]. This alteration allows the detection of AD-related protein biomarkers in the bloodstream, albeit at lower concentrations compared to those found in the CSF, as reported for the core AD proteins Aβ [125] and t-tau, which are approximately 100 times less concentrated than in CSF [126]. There are two valid reasons to investigate the presence of AD biomarkers in plasma. The first is that blood samples can be obtained through minimally invasive procedures, thus avoiding the need for lumbar puncture. The second is that plasma biomarkers could be measured at a relatively low cost once standardized analytical methods are established. Indeed, many laboratories worldwide base clinical diagnoses on plasma samples, as in the case of C-reactive protein levels used to assess coronary disease [127]. However, blood represents a highly complex matrix, posing several challenges for proteomics biomarker detection, and MS analysis remains particularly demanding. One major issue is the wide dynamic range of plasma proteins. Albumin, for example, is present at concentrations up to 10 orders of magnitude higher than the rarest proteins [128] and accounts for about 50% of the total plasma protein content. Approximately 22 proteins make up 99% of the total plasma protein mass. The detectability of low-abundance proteins can be improved by depleting high-abundance proteins to enrich the sample, although this carries the risk of losing low molecular weight proteins that bind to albumin [129]. Some proteins may be produced exclusively in the CNS, whereas others are also expressed in peripheral tissues or organs. In the latter case, differential expression could reflect systemic effects unrelated to AD, making it particularly difficult to identify AD-specific mechanisms. Further, the possible comorbidities, such as vascular problems or systemic inflammation can make really difficult the interpretations of the data.

Importantly, many plasma biomarker studies remain at exploratory or early validation phase, often relying on heterogeneous analytical platforms, small cohort sizes, or highly selected populations. These methodological limitations restrict the generalization of the findings and indicate that most plasma biomarkers should still be considered experimental rather than clinically applicable. Greater standardization of pre-analytical procedures, longitudinal validation and harmonization of MS, and immunoassay methods are essential before integrating these markers into routine diagnostic workflows.

### 5.1. Aβ as Plasma Biomarkers in AD

Earlier studies on AD plasma biomarkers focused on molecules known to be related to AD etiology, namely APP, Aβ, and p-tau [130]. Aβ fragments are particularly difficult to evaluate because they bind to plasma proteins and, partly, are produced by platelets, introducing variability in concentration measurements [131]. Furthermore, Aβ40, Aβ42, and tau are present at much lower levels in blood, and, given that blood is a highly complex matrix, it can be very challenging to detect proteins involved in AD etiology and progression. The high abundance of antibodies may lead to false results [132], and protein biomarkers are subject to proteolytic degradation in the liver or may be metabolized and cleared from circulation [133]. One of the classical CSF biomarkers, the Aβ42/Aβ40 ratio, shows a similar decrease in the plasma of AD patients, indicating a sequestration of Aβ inside plaques.

Most studies have reported a reduction in this ratio in MCI and AD patients, as well as in individuals with progressive MCI or at risk of developing MCI and AD. However, in a few cases, the data have been partially contradictory, showing an increase in the ratio associated with a higher risk of AD development [129]. Since 2018, immunoprecipitation coupled with MS has enabled reliable quantification of the Aβ42/Aβ40 ratio, although the decrease averages at about 50% in CSF and only 10–15% in blood [134]. The higher sensitivity reached in the evaluation of Aβ42/Aβ40 ratio in the plasma with immunoprecipitation techniques has allowed to discriminate amyloid positive patients with more than 85% accuracy [135]. Aβ42/Aβ40 ratio has been found to achieve accuracy levels similar to those obtained from PET imaging for amyloid deposition [136].

Despite these encouraging results, the interpretation of plasma Aβ data remains challenging due to platform-dependent variability, partial overlap between diagnostic groups, and susceptibility to pre-analytical biases (e.g., anticoagulant type, storage time). For these reasons, plasma Aβ testing—although promising—can not be considered equivalent to CSF or PET measures in clinical decision-making.

### 5.2. p-tau as Plasma Biomarkers in AD

Following advances in plasma Aβ detection, MS-based assays have also enabled the identification of p-tau in plasma samples [137], which is now recognized as a specific biomarker for the early stages of AD [138]. The relevance of plasma p-tau compared with CSF p-tau has been highlighted by recent studies exploring different phosphorylation states of the protein [139]. In presymptomatic individuals, plasma p-tau levels increase more than ten years before symptom onset, and p-tau increase is associated with rapid cognitive decline and hippocampal atrophy [140,141,142]. Further p-tau markers discriminate between AD patients and controls more effectively than other plasma biomarkers, such as the Aβ42/Aβ40 ratio and NfL [143].

Different p-tau fragments can be detected in plasma as putative biomarkers and show high diagnostic accuracy. Plasma p-tau181, p-tau231, and p-tau217, phosphorylated on threonine residues, are excellent indicators of the symptomatic stage of AD, increasing with disease severity and showing strong associations with amyloid and tau pathologies [144]. Plasma p-tau181 levels are higher in AD patients compared to control individuals and correlate well with CSF p-tau181. Levels increase with disease progression and serve as a reliable marker for distinguishing AD dementia from non-AD dementias [145]. MS analysis of different p-tau forms, namely p-tau181, p-tau217, and p-tau205, has focused on their utility in monitoring AD progression over time [137]. Recent data indicate that elevated plasma p-tau181 is associated with future deposition of Aβ plaques in various brain regions, suggesting its potential use as a biomarker for amyloid accumulation [146]. Among these isoforms, p-tau217 has emerged as particularly promising. It shows a several-fold increase in symptomatic AD patients and demonstrates superior performance in distinguishing AD from non-AD neurodegenerative disorders, with diagnostic accuracy comparable to CSF biomarkers and amyloid PET imaging [139]. Importantly, p-tau217 appears to increase earlier in the disease continuum than p-tau181, highlighting its potential for preclinical detection and for stratifying individuals at risk. The high prognostic value of p-tau217 makes it one of the best marker of clinical relevance [147]. Plasma p-tau231 has also been identified as an early marker, potentially rising during the initial phases of amyloid deposition before clear tau aggregation becomes detectable. This makes p-tau231 especially relevant for identifying individuals at preclinical and prodromal stages of AD, when therapeutic interventions may be most effective.

Nevertheless, despite their excellent performance across several cohorts, plasma p-tau assays still face key challenges, including assay-dependent variability, limited availability of harmonized reference materials, and possibly confounding facts from comorbidities or demographic factors. Therefore, while plasma p-tau biomarkers represent a major advance, they should currently be interpreted as complementary tools rather than standalone diagnostic tests. In conclusion, cross-platform standardization, reproducible studies across diverse populations and long-term clinical validation are required before these biomarkers can be integrated into routine practice.

### 5.3. Other Plasma Biomarkers

NfL levels increase in the prodromal stage of AD, increasing in bloodstream following axonal damage. However, its levels are also associated with various neurodegenerative diseases, making NfL alone an unspecific biomarker for the diagnosis of AD [120].

The glial fibrillary acidic protein (GFAP), released from astrocyte cytoskleton, shows elevated blood concentrations not only in AD dementia, but also in other neurodegenerative and non-neurodegenerative conditions [148,149,150]. Thus, it can not be considered a specific biomarker for AD. However, some cues point to its employment as AD biomarker. GFAP can discriminate between Aβ-positive and Aβ-negative subjects [151]. GFAP levels correlate with Aβ pathology [152] and with declined cognitive abilities [153]. Then, GFAP should be included in the blood biomarker panel as a useful indicator for detecting reactive astrogliosis and Aβ pathology. The combination of NfL, GFAP, with p-tau181 and p-tau217 provides good predictive properties for dementia and AD development [38].

Still, the majority of these markers (e.g., Nfl, GFAP, IL-10, inflammatory markers) lack disease specificity and are influenced by systemic conditions, age, and comorbidities. Their clinical utility is therefore limited unless used in combination with more AD-specific indicators such as p-tau isoforms. Careful methodological validation is necessary to avoid over-interpretation of preliminary associations.

Beyond the classical AD biomarkers above reported, a recent meta-analysis highlighted six putative biomarkers consistently reported across independent cohorts [154].

Alpha-2-macroglobulin (α2M), a component of the innate immune system is linked to vascular dysfunction and to up- or downregulated proteins of the complement cascade [36,155]. Its role in inhibiting coagulation could delay the repair of endothelial cells in the BBB, thereby facilitating the entry of pro-inflammatory molecules into the brain [156]. α2M is associated with CSF concentrations of tau and p-tau proteins, and its serum concentration correlates with cognitive decline compared to healthy individuals [157].

Apolipoprotein A1 (ApoA-1), the main component of high-density lipoproteins (HDLs) derived from the periphery, crosses the BBB moving to brain parenchyma where it plays a critical role in preserving cerebrovascular integrity by modulating Aβ aggregation [158]. Serum from AD patients shows lower levels of ApoA-1 compared to normal individuals [159], mirroring its decrease in CSF and correlating with disease severity [160].

Afamin is a specific binding protein transporting vitamin E across the BBB to the brain, where it has neuroprotective activity against oxidative mechanisms. Afamin is downregulated in AD patients, potentially rendering the brain more vulnerable to oxidative stress [161].

Fibrinogen-γ chain-A has an important role in fibrin polymerization and platlet aggregation [162]. As potential biomarker, it may be associated with increased vascular damage, consistently with previous observations for fibrinogen in CSF [163] and fibrinogen isoforms detected in plasma, using 2DE gel electrophoresis combined with MALDI TOF/TOF-MS [154].

Pancreatic polypeptide (PP) is upregulated in plasma samples from MCI and AD patients, and it may predict underlying AD through association with CSF AD biomarkers. PP levels correlate with the level of CSF Aβ and the ratio of tau/Aβ42, reflecting the neuronal loss and alterations of the BBB [164].

The plasmatic increase of insulin-like growth factor binding protein 2 (IGFBP2) has been associated with a major risk of developing AD [165], and IGFBP2 may have a specific role in AD pathophysiology. High levels of plasma IGFBP2 are associated with the AD-like pattern of brain atrophy [166].

Overall, while these emerging plasma biomarkers offer valuable insights into AD pathophysiology, their use into routine clinical practice requires careful validation through longitudinal, multicentric studies with harmonized analytical protocols. Emphasizing the preliminary nature of these findings strengthens the scientific rigor of the manuscript.

A summary of the plasma biomarkers of AD and their main features are shown in Table 2.

## 6. Other Protein Biomarkers in AD

The substantial diversity of differentially expressed proteins identified across multiple proteomics studies confirms the complexity of AD etiology and pathology, highlighting its multifaceted nature. Consequently, novel biomarkers research into novel and emerging biomarkers goes on in a constant effort to improve AD diagnosis and prognosis.

High-resolution MS combined with tandem mass-tags-based multiplexing and immunodepletion techniques applied to 5 control and 5 AD patient samples identified 139 out of 2327 differentially expressed proteins, including t-tau, neuronal pentraxin-2 (NPTX2), GFAP, neuronal cell adhesion molecule-1 (NCAM1), pyruvate kinase M (PKM), and tyrosine 3-monooxygenase/tryptophan 5-monooxygenase activation protein gamma (YWHAG). While t-tau and GFAP are already well-established markers of neurodegeneration and astroglial activation, the identification of NPTX2, PKM, and YWHAG points to newer pathways that may complement existing diagnostic tools. NPTX2, in combination with PKM or YWHAG, gives out results with high accuracy of 0.935 and 0.933, respectively [167]. NPTX2 interacts with glutamatergic receptors and acts as modulator of synaptic activity, facilitates excitatory synapse formation, and contributes to brain plasticity, learning, and memory. Its downregulation, although not fully validated as a clinical biomarker, is increasingly recognized as a promising indicator of early synaptic dysfunction, and it may predict memory loss and brain atrophy over time [168].

Immunodepletion, in a multiplex tandem mass-tag labeling study allowed the identification of 225 downregulated and 303 upregulated proteins out of 2875 profiled in CSF samples, from 20 controls and 20 AD patients. Proteins, such as tau, NPTX2, and NCAM1, which show consistent dysregulation across studies, are considered stronger candidates, and were grouped into five panels reflecting dysregulation in synaptic activity, vascular function and coagulation, cellular structure, myelination, as well as inflammatory and metabolic pathways [169].

A multiplex proteomics study of CSF and blood biomarkers across different cohorts identified protein alterations linked to inflammation, apoptosis, and other biological processes associated with Aβ42 and tau deposition. Several chemokines, interleukins, and immune markers were altered in AD patients, but their diagnostic utility remains largely exploratory due to modest reproducibility across cohorts. Caspase 8, implicated in synaptic plasticity, amyloid processing, and microglial pro-inflammatory activation, was elevated in both CSF and blood of AD patients, Although mechanistically relevant, its role as a biomarker remains speculative and therapeutic implications require further validation [170].

Several proteins involved in synaptic activity are altered in AD CSF. Presynaptic synaptosomal-associated protein 25 (SNAP-25) shows higher CSF concentrations in various neurodegenerative disorders and in early-stage AD. Although it is not specific to AD diagnosis, SNAP-25 is considered a more established marker for monitoring synaptic degeneration and disease progression [171]. Similarly, synaptotagmin-1 (SYT-1) may serve as marker of progressive cognitive decline, since it shows higher levels in MCI patients progressing to AD [172].

Neurogranin is a post-synaptic protein abundantly expressed in the brain, where it is involved in synaptic plasticity and long-term potentiation by regulating intraneuronal calcium signaling. Neurogranin levels have been found increased in CSF, but decreased in blood plasma exosomes of patients with AD and MCI-AD [173]. It is one of the more validated emerging biomarkers of synaptic degeneration, although its specificity is still under study [174].

Wang and colleagues [175] have conducted an integrated ultra-deep proteome analysis, identifying 37 proteins as potential AD markers across cortex, CSF, and serum. By integrating multiple proteomes and employing MS techniques within a systems biology framework, they found that 59% of these proteins were involved in mitochondrial dysfunction, supporting the hypothesis that mitochondrial alterations may contribute to AD pathogenesis [176,177]. While these findings provide valuable mechanistic insights, most of these mitochondrial-related markers remain at the discovery stage and require extensive replications. Consistent with these findings, decreased levels of mitochondrial thioredoxin-dependent peroxide reductase (PRDX3), a protective antioxidant enzyme, were observed in AD CSF, suggesting mitochondrial dysfunction and an imbalance in redox homeostasis [178].

Mannosylated-glycan transferrin (Man-Tf), a post-translationally modified transferrin isoform produced by cortical neurons, is elevated in AD CSF, likely due to endoplasmic reticulum oxidative stress, as detected by ultra LC-MS. Man-Tf strongly correlates with p-tau, consistent with hippocampal neurons co-stained for both proteins, suggesting that combined measurement of p-tau and Man-Tf may offer a promising, though still under investigation, biomarker pair for MCI and AD [179]. A summary of putative biomarkers under evaluation for AD is shown in Table 3.

A five-protein biomarker panel has been proposed by Naveed et al. [180] as a signature for AD diagnosis, performing with 90.1% sensitivity, 87.9% precision, and 84.2% specificity. The panel included S100 calcium binding protein A9 (S100A9), directly linked to AD [181], alpha-globulin 1, and endothelial cell adhesion molecules CD84 and CD226. This five-protein panel can diagnose AD and differentiate from other neurological diseases, but requires validation in larger and more diverse cohorts before it can be considered clinically established. This supports the notion that a multi-panel approach may be a powerful tool to improve diagnostic accuracy.

A recent study combined a multiple reaction monitoring (MRM)-MS approach with machine learning, enabling the quantification of 125 plasma proteins and the prediction of disease progression. Afamin, ApoE, biotinidase, and paraoxonase/arylesterase showed significant decreases, alongside other proteins previously reported as blood-based biomarker candidates [182].

Although these findings highlight the potentiality of high-throughput plasma proteomics, most identified biomarkers are still preliminary and require rigorous validation.

In conclusion, while many protein biomarkers have been associated with AD, only a few have progressed toward clinical utility. The majority remain exploratory and their specificity and sensitivity must be established through larger, longitudinal, and multi-center studies before reliable diagnostic implementation is possible (resuming evidences are listed in Table 4).

## 7. AD Biomarkers of Other Biological Fluids

As mentioned before, lumbar puncture to collect CSF samples is a very invasive procedure. Blood is more accessible, but requires specific procedures as well, needing to be carried out by technical personnel. For these reasons, there is wide interest in finding new sources of possible AD biomarkers in biological fluids such as saliva and urine. Both biofluids are easy to collect, with no invasive practice, and are thus much more viable for mid-life population.

### 7.1. Saliva Biomarkers

Saliva has been proved rich in biomarkers for the detection of different CNS diseases due to the excretion of central proteins into it [183]. The fingerprint AD biomarkers, Aβ peptides and tau proteins, are found in saliva samples due to the fact that brain-derived peptides are transported to the periphery via the BBB and can be easily collected from the oral cavity. The study of Aβ peptides in saliva fluid still shows conflicting results. Some studies on salivary Aβ42 have led to the detection of an increase in AD patients versus control subjects [184,185] and in AD patients versus MCI prodromic stage [185], making Aβ42 a valid indicator for the detection and monitoring of the disease. On the contrary, an immunology multiplex assay has evaluated a lower level of the peptide in AD patients compared to healthy subjects [186] and non-significant differences in other studies [187,188]. A similarly unclear result pattern has been retrieved for Aβ peptide with levels in AD patients higher than control [189] and no difference detectable in other studies [190,191].

Tau proteins reach the saliva environment crossing the BBB [192], reflecting the level changes occurring in CNS. p-tau measures have yielded contrasting results, as already mentioned for previous Aβ peptides. Some studies conducted with different techniques report an increase of p-tau in AD patients compared to healthy individuals [188,193]. On the contrary, similar procedures have led to undetectable differences in the two groups [186,191]. t-tau and the p-tau/t-tau ratio similarly have shown opposite outcomes [186,188,191]. Although CSF and plasma Nfl levels are higher in AD patients, as reported above, no difference has been found in salivary samples from AD and healthy subjects [194].

Neuroinflammation, as stated above in this paper, is an important component in AD etiology due to release of multiple cytokines from glial cells. Salivary samples exhibit unclear results with significant decrease in interleukin-1 (IL-1), interlukin-6 (IL-6), and tumor necrosis factor α (TNF-α) in AD patients and opposite results for cyclooxygenase-2 (COX-2), caspase-8, interleukin 1β (IL-1β), and metalloproteinase-9 (MMP-9) [195]. Further, GFAP, which shows higher plasma levels in AD patients as in other neurodegenerative diseases (see Other plasma biomarkers), has resulted decreased in salivary samples with a decreasing trend as AD proceeds [193].

Lactoferrin is a salivary glycoprotein involved in iron absorption that also acts as an antimicrobial agent, which is found in Aβ plaques and NFTs following its rapid transfer across the BBB. Lactoferrin salivary levels decrease in AD patients and are correlated to CSF Aβ42 and t-tau, suggesting its possible employment as AD biomarker [196]. In contrast, another study did not observe any difference between AD patients and controls [194].

Proteomics analysis of saliva fluid has identified different metabolites with different expression levels between AD and healthy cohort participants, which could be considered for future AD evaluation.

In conclusion, although many studies have been conducted to identify possible AD biomarkers in an easy collected biofluid, many doubts remain due to the widespread lack of specificity and reliability. Many problems arise in identifying a valid biomarker for AD. First, it would be necessary to analyze patients at the same stage of the disease to rule out variations in biomarkers related to disease rate progression. Second, many biomarkers are not specific to AD and therefore require more precise validation. Thirdly, some biomarkers, especially those related to inflammation, may be correlated with non-manifest inflammatory events that alter the assessment. It should also be considered that the oral environment is strongly influenced by possible pathological events related to chewing, oral flora, possible dental interventions, and medication intake that can significantly alter the results. Finally, the collection and, above all, the preservation of samples can present problems that affect the results obtained.

Anyway, the analysis of saliva is a promising pathway that needs to be improved in terms of analysis of patient cohorts at different disease stadiation (MCI to AD), common and standardized collecting and analyzing procedures, as well as ability to exclude confounding factors.

### 7.2. Urine Biomarkers

Urine is a very accessible biofluid, which can be collected by the patient in the home environment and brought to a lab for analysis, without any personnel intervention. Despite this positive cue, urine contains very low amounts of proteins, making protein detection more difficult. One of the possible approaches to finding novel AD biomarkers in urine fluid is the identification of differentially expressed genes in brain samples, which eventually confirm the findings in the levels of urinary proteins encoded by the same genes.

Following this scheme, a computational analysis has helped in the identification of 2754 differentially expressed genes, 559 of which are coded for urine proteins. Osteopontin, gelsolin, and insulin-like growth factor binding protein 7 (IGFBP7) resulted in differentially expressed genes and have been proposed as potential urine protein biomarkers for AD [197]. Alzheimer-associated neuronal thread protein (AD7c-NTP) increases in AD urine samples, but the results were controversial and specificity and sensitivity are still lacking [198].

High levels of p-tau phosphorylated on serine 396 (p-S396) have been detected in urinary exosomes of AD patients [198].

At present, urinary AD biomarkers still have low specificity and sensitivity because standardized collecting and storing procedures are not available. For this reason, any novel putative urine biomarkers should be validated by other samples, after having excluded side events due to renal dysfunction. The best approach now is to use urine samples to provide a first screening of the disease, followed by analysis of other biofluids such as plasma or CSF to provide a higher reliability.

## 8. MicroRNAs and Extracellular Vesicles in AD Biomarker Research

### 8.1. microRNAs

MicroRNAs (miRNAs) are small non-coding RNAs conserved across different species, with a wide tissue/cell expression. miRNAs have important regulatory roles, and their deregulation has an important impact on organism health. miRNAs in CNS guide neurogenesis, neurodevelopment, and neuronal differentiation [199], and their deregulation is involved in CNS disorders, suggesting that the aberrant expression of miRNAs can be properly used in disease biomarker research [200,201].

Well-characterized miRNAs exhibited a significant downregulation in the gray matter of AD patients, correlating with the density of amyloid plaques and probably contributing to AD pathogenesis [202]. Different miRNAs have been found deregulated in AD with strong implications in APP processing, neuroinflammation, and tau phosphorylation, and some of them seem to be involved in more than one process, indicating that they can also mediate cross-talking among the different pathological events underlying AD [203]. The combination of 2–4 CSF miRNAs can distinguish AD patients from controls with high specificity (75–82%). The combination of different miRNAs can distinguish AD from FTD [204], Parkinson’s disease, and vascular dementia [205] with high sensitivity and specificity [206]. Moreover, miRNAs are able to predict MCI individuals at risk of developing AD with a 95.3% sensitivity. The analysis of miRNAs is a novel area that offers a greater capability for diagnosis, prognosis, and therapeutic progress. Despite these positive cues, work must still be done to set correct reference values and unambiguous outcomes.

### 8.2. Extracellular Vesicles

Extracellular vesicles are small particles delimited by lipid bilayers, containing proteins, lipids, and genetic materials. They are released by cells and play crucial roles in intercellular communication and various physiological processes. Their study has gained much attention not only due to their potential as biomarkers for disease diagnosis, but also for therapeutic targets and drug delivery systems. In AD, the extracellular vesicles derived from the CSF contain high levels of Aβ peptides, contributing to toxicity in vitro [207] and in vivo [208]. Vesicles released from microglia contain tau proteins that promote disease progression [209].

Extracellular vesicles can thus be potential biomarkers reflecting the stadiation of AD [210] and can be collected from plasma samples due to the breakdown of the BBB in AD. Further, the vesicles-based therapies could be a more efficient and functional delivery system to reach and pass the BBB in the attempt to slow down the disease progression.

## 9. Conclusions and Future Directions

Molecular biomarkers are molecules that can be used to discriminate between healthy and diseased conditions. In general, such molecules can have diagnostic value for detecting pathology, a staging value for disease progression, as well as prognostic value for predicting the final outcome. They can also be used to monitor clinical responses. Protein biomarkers have received widespread attention due to the application of proteomics techniques, as proteins are highly sensitive and can be detected in very small sample amounts, enabling early diagnosis. Furthermore, proteomics can identify protein post-translational modifications, which may play pivotal roles in disease onset and progression. The application of proteomics to AD research has expanded expectations regarding early diagnosis and prognosis. Proteins are collected from biofluids, with CSF and blood being the most studied. Significant work has been carried out to discover proteome-based biomarkers in biofluids, establishing important milestones in AD knowledge. In addition to Aβ42, the Aβ42/Aβ40 ratio, and p-tau as established CSF biomarkers, many other candidates have been proposed to improve AD monitoring. However, it must be recognized that CSF-based proteomics have several limits for patient handling, firstly because of the invasive approach in sample collection with the lumbar puncture. Blood-based proteomics overcomes this hard point, offering a less invasive sample collection, but showing other challenges such as protein cross-identifications in case of comorbidities, as well as sample quality and difficult detection for low-abundance proteins. Unfortunately, most of these novel biomarkers are currently available only as research tools and are not yet implemented in routine clinical practice. To move towards a more sensitive and specific diagnosis and prognosis for AD many points need to be set.

First of all, it is necessary to establish common protocols, which must be undertaken worldwide, setting the same procedures for sample collection, storage, and analysis. Alongside this point, larger cohorts of healthy individuals, patients affected by non-AD neurodegenerative conditions, and AD patients at different stages must be compared to improve the detection accuracy of biomarker panels.

The use of AD biomarkers is still evolving and requires more time to achieve clinically validated molecules with robust diagnostic and prognostic value. In the recent years, machine learning has proven to be a strong aid for the identification of specific biomarkers and to set therapeutical approaches by analyzing protein interactions. Looking forward, the integration of proteome-based biomarkers with other diagnostic modalities, such as neuroimaging and genetic testing, holds significant promise for improving early and accurate detection of AD [211].

Proteome-based biomarkers, combined with advanced MS techniques and machine learning tools, should aim to develop protein platforms that include both highly specific and less specific AD markers, which may escape detection with current data analysis methods. These platforms could lead to very early diagnosis, provide real-time information on disease progression, offer reliable indicators for medical treatment, and offer better knowledge of the underlying molecular mechanisms of the disease.

AI and machine learning approaches will be particularly valuable not only for analyzing proteomics data but also for integrating multiple data sources, including neuroimaging, genomics, and other omics datasets, to construct comprehensive biomarker panels. This integrative strategy can enhance predictive accuracy, facilitate personalized therapeutic interventions, and stratify patients based on subtypes of AD [212]. For example, integrated genomic, transcriptomic, and proteomic analyses have already been applied in large consortia such as Alzheimer’s Disease Neuroimaging Initiative (ADNI), identifying multi-omic markers associated with disease stages [213]. Moreover, recent multi-cohort proteomics studies of CSF using data-independent acquisition MS, coupled with machine learning, have identified subtype specific protein classifiers of AD that differentiate between Aβ^+^/tau^+^ and Aβ^+^/tau^−^ individuals [213]. Technological advances in MS (e.g., tandem-mass-tag TMT, data-independent acquisition DIA, and high sensitivity instruments) and affinity-based platforms (such as Olink and SomaScan) now allow for deeper and more reproducible proteome coverage [214]. Post-translational modifications, accessible via MS, add another layer of biomarker richness relevant for neurodegeneration [215]. Integrating proteomics with genomics via protein quantitative trait locus (pQTL) analyses can link genetic risk factors to changes in protein expression, helping to elucidate causal mechanisms [214]. Furthermore, AI-based models applied to neuroimaging data (e.g., MRI, PET) have shown high performance in early AD classification and progression prediction [216]. Machine learning models that fuse neuroimaging and proteomic/genetic data are increasingly being explored and may overcome current limitations of single-modality approaches. Such a “multi-modal” paradigm is poised to revolutionize diagnosis, prognosis, and the personalization of therapy in Alzheimer’s disease. Ultimately, such integrative approaches will advance our understanding of one of the most debilitating neurodegenerative diseases and accelerate the translation of biomarker discoveries into clinical applications.

## Figures and Tables

**Table 1:** CSF biomarkers and proteomic findings in Alzheimer’s disease.

Biomarker		Key Features & Findings	Diagnostic Value	Limitations
**Amyloid-related biomarkers**	Aβ40	Soluble isoform of Aβ, normally present [7]	Used as denominator for Aβ42/Aβ40 ratio [73,74]	Alone not predictive [70]
	Aβ42	Insoluble, hydrophobic, reduced in CSF of AD patients due to plaque sequestration; main plaque component [7]	Strong predictor of progression to MCI/AD; correlates with amyloid plaque load [66,67]	Reduced also in other pathologies (e.g., bacterial meningitis) [68]; depends on total Aβ pool [70]
	Aβ42/Aβ40 ratio	Improves accuracy over Aβ42 alone [73,74]	Better discrimination between AD and non-AD dementia; detectable before cognitive impairment [66,67]	Requires precise quantitation [72]
	Total Aβ	Similar in healthy vs. AD individuals [70]	Not useful for AD diagnosis	No discriminative value
**Tau-related** **biomarkers**	t-tau	Increases due to neuronal loss; Correlates with proteins linked to plasticity & BBB dysfunction [80,81,82]	-Gold standard biomarker; predicts progression from MCI to AD [67]	Elevated in other neuronal damage, not AD-specific [82]
	p-tau (p-tau181, p-tau231, p-tau217)	Hyperphosphorylated tau isoforms; linked to NFT formation; p-tau181 validated in clinical use [87]	Sensitive & specific for AD; combined with Aβ42 for strong diagnosis [89,90]	Some overlap with other tauopathies [89]
**Combined markers**	Low Aβ42 + high t-tau/p-tau	Reflects amyloid plaques + NFTs [67]	Strong biomarker panel for AD diagnosis; High accuracy [89,91,93]	Requires lumbar puncture & standardized assays [91]
**Other CSF biomarkers**	Neurofilament light (NfL)	Marker of axonal degeneration; elevated in AD and other neurodegenerative diseases [20]	Useful to monitor disease progression; NfL/Aβ42 ratio predicts atrophy & cognitive decline [20]	Not AD-specific [20]
**Inflammatory/Oxidative** **stress markers**	YKL-40 (CHI3L1)	Astrocyte-derived protein, reflects neuroinflammation; associated with cortical atrophy & progression [110,111]	Potential prognostic biomarker; could guide anti-inflammatory therapies [112]	Elevated in several inflammatory disease; not AD-specific [112]
	sTREM2	Microglia-related receptor; correlated with t-tau and p-tau181 [109]	Candidate biomarker of microglial activation in AD [109]	Also increased in other neurodegenerative disorders [106,107]
	Osteopontin	Inflammatory marker, may potentiate immune response [113]	Possible biomarker for AD immune response [113]	Elevated in several inflammatory disease; not AD-specific [113]
	Cytokines (IL-10, MIF, MCP-1)	Altered levels in AD vs. non-AD patients [20]	Candidate biomarkers of neuroinflammation [20]	Require further validation [20]
**Proteomics &MS-based** **discovery**	LC-ESI-MS, MALDI-TOF-MS	First identified Aβ fragments (before 1992) [76,77]	Enabled discovery of AD-linked proteins [76,77]	Limited by early database availability
	Post-2003 (post-HGP)	Expanded proteome databases → full-scale proteomic analyses [36,47]	Allowed targeted assays for Aβ, tau, and new proteins [36,42]	Variability across studies [56]
	Recent MS-based studies	Identified 40+ up/down-regulated proteins, e.g., FABP3, YKL-40 [50,51,52]	Advanced biomarker discovery; classification of AD vs. non-AD [36]	Cohort variability, reproducibility issues, non-specificity [50,52]

**Table 2:** Plasma biomarkers in Alzheimer’s disease.

Biomarker	Key Features & Findings	Diagnostic Value	Limitations
General context	- AD damages the blood–brain barrier, allowing proteins (Aβ, tau) to [122,123] - Blood sampling: minimally invasive, low-cost potential if standardized [127,128] - Challenges: complex plasma matrix, high-abundance proteins (e.g., albumin, 22 proteins = 99% of plasma weight), risk of losing low-abundance proteins during depletion [127,128]	Advantage: non-invasive, scalable for clinical use [126]	- Limitations: proteomic detection is difficult; - Possible confounding from peripheral protein production, degradation and metabolism in blood [131]
Aβ (Amyloid-β)	- Aβ fragments bind plasma proteins, partly platelet-derived (measurement disturbance) [131] - Very low concentrations in plasma [142] - Aβ42/Aβ40 ratio: decreased in AD and MCI, but some contradictory results [134,135] - MS + immunoprecipitation (since 2018): improved detection [134] - Plasma decrease less pronounced than in CSF (10–15% vs. ~50%) [134]	Aβ42/Aβ40 ratio: recognized as early AD marker with high accuracy [134,135]	- Still difficult to detect reliably [134,135] - Antibody interference and degradation possible [134] - Less discriminative than p-tau [132]
p-tau (phosphorylated tau)	- Plasma p-tau (p-tau181, p-tau217, p-tau231) shows strong correlation with CSF and PET markers [136,138] - p-tau increases >10 years before symptoms [136] - p-tau181: correlates with AD progression, dementia, amyloid plaques [140,145] - p-tau217: superior accuracy, earlier rise than p-tau181, discriminates AD vs. non-AD with PET/CSF-level accuracy [137,139] - p-tau231: rises early during amyloid deposition, relevant for preclinical detection [147] - Combinations (e.g., p-tau217+p-tau231) improve sensitivity and specificity [139]	- Excellent diagnostic accuracy, minimally invasive [138,139] - Outperforms Aβ ratio and NfL in discrimination [139] - Transformative potential, track progression, stratify risk, enable early/preclinical detection [139,140,141]	Need for assay standardization, population validation, reference thresholds [140,152]
NfL (Neurofilament light)	- Increases in prodromal AD and elevated in many neurodegenerative diseases [120,121]	Sensitive but not specific for AD [120,121]	Best used in biomarker panels [150]
GFAP (Glial fibrillary acidic protein)	- Elevated in CSF and plasma in AD and other disorders [149,151,153] - Correlates with worse AD outcomes [151] - Negatively correlated with Aβ42/Aβ40 [152]	Not AD-specific, but valuable marker of astroglial activation [148,151]	Recommended use in biomarker panels [150,151]
α2-Macroglobulin (α2M)	- Elevated in AD patients, correlates with CSF p-tau and cognitive decline [157] - Linked to vascular dysfunction and complement cascade [155,157] - Stage-dependent: downregulated in pre-symptomatic stages [157]	Reflects systemic inflammation and vascular effects [157]	May contribute to BBB dysfunction [155,157]
Apolipoprotein A1 (ApoA-1)	- Downregulated in plasma and CSF of AD patients, likely due to Aβ binding [158,160]	Suggests role in lipid metabolism and amyloid clearance [159]	
Afamin	- Transports vitamin E (antioxidant) and downregulated in AD plasma [117,118]	Reduced antioxidant defence and higher vulnerability to oxidative stress [117,118]	
Fibrinogen-γ-chain	- Upregulated in plasma and CSF [162,163] - Associated with vascular damage [162,163]	Marker of vascular dysfunction in AD [162,163]	
Pancreatic polypeptide (PP)	- Consistently upregulated in several cohorts [164,165]	Potential biomarker [164]	Mechanism not defined [164]
IGFBP2 (Insulin-like growth factor binding protein-2)	- Involved in neuronal energy metabolism - Interacts with tau and Aβ in CSF - Associated with cognitive decline [117,118]	May mediate systemic inflammatory/energy processes affecting AD pathology [117,118]	

**Table 3:** Proteomics biomarkers under evaluation for Alzheimer’s disease.

Biomarker	Biological Role	Finding in AD	Relevance
t-tau	Microtubule-associated protein	Increased	Core AD biomarker [167]
NPTX2 (Neuronal Pentraxin-2)	Modulates synaptic activity, excitatory synapse formation	Downregulated	Predicts memory loss, brain atrophy, linked to cognitive dysfunction [168]
GFAP (Glial Fibrillary Acidic Protein)	Astrocyte marker	Altered levels	Marker of astroglial activation [169]
NCAM1 (Neuronal Cell Adhesion Molecule-1)	Neuronal adhesion, synaptic plasticity	Dysregulated	Impaired synaptic connectivity [169]
Glucose metabolism proteins	Energy metabolism	Increased in CSF	Reflect brain tissue release, early metabolic dysfunction [167]
Cannabinoid receptor 1	Endocannabinoid signaling	Correlated with Aβ42	Potential therapeutic target [169]
Neuroendocrine convertase 2	Neuropeptide processing	Correlated with Aβ42	Supports neuroendocrine involvement [169]
Somatostatin	Neurotransmitter	Correlated with Aβ42	Linked to cognitive function [169]
SNAP-25 (Synaptosomal-associated protein 25)	Presynaptic vesicle fusion	Increased (early AD CSF)	Early synaptic dysfunction marker [171]
SYT-1 (Synaptotagmin-1)	Presynaptic calcium sensor	Increased (MCI → AD)	Marker of progression [169]
Neurogranin	Postsynaptic plasticity protein	Increased in CSF, correlated with tau/p-tau	Reflects synaptic loss, predictor of decline [173,174]
Neuromodulin (GAP-43)	Presynaptic plasticity	Downregulated	Indicator of impaired cognition [169]
PRDX3 (Thioredoxin-dependent peroxidase)	Antioxidant enzyme, mitochondrial protection	Decreased	Indicates mitochondrial dysfunction, oxidative imbalance [175]
UCHL1 (Ubiquitin C-terminal hydrolase L1)	Protein degradation	Altered	Biomarker of proteostasis dysfunction [172]
FABP3 (Fatty Acid Binding Protein 3)	Lipid metabolism	Altered	Diagnostic potential [169]
PKM (Pyruvate Kinase M)	Glycolysis enzyme	Increased	Marker of glucose metabolism alteration, neurodegeneration [167]
Caspase 8	Apoptosis, synaptic plasticity, amyloid processing	Increased (CSF & blood)	Potential therapeutic target (inhibition may aid survival) [169]
JAM-B (Junctional Adhesion Molecule-B)	Synaptic adhesion	Downregulated	Associated with cognitive decline [169]
MMP9/MMP10 (Matrix Metalloproteinases)	Extracellular matrix remodeling, Aβ degradation	Upregulated	Correlates with cognition, potential role in Aβ clearance [169]
Man-Tf (Mannosylated-glycan Transferrin)	Modified transferrin, neuronal origin	Increased, correlates with p-tau	Proposed combined biomarker (p-tau + Man-Tf) for MCI/AD [179]
Chemokines, Interleukins, Immune markers	Inflammatory signaling	Altered	Reflect neuroinflammation in AD [180,181,182]
Mitochondria-related proteins	Energy metabolism	59% of novel markers linked to mitochondrial dysfunction	Supports mitochondrial hypothesis of AD [175,176,177]

**Table 4:** Protein biomarkers in AD: Established, Emerging, and Speculative.

Category	Biomarkers	Rationale for Classification	References
**Established**	- t-tau - p-tau - GFAP - Neurogranin (partially established, especially in CSF) - SNAP-25 (for monitoring synaptic degeneration)	Biomarkers repeatedly validated across multiple cohorts. Some (tau, GFAP) are already used in diagnostic protocols or in international multicenter studies (e.g., ADNI). SNAP-25 and neurogranin are widely reproduced as indicators of synaptic degeneration.	[167,169,171,173,174]
**Emerging**	- NPTX2 - NCAM1 - SYT-1 - PKM - YWHAG - Man-Tf - PRDX3 - 5-protein panel (S100A9, α-globulin 1, CD84, CD226, etc.) - Afamin, plasma ApoE (in MRM-MS predictive models)	Biomarkers with growing evidence, often replicated in multiple proteomic studies, but not yet integrated into diagnostic criteria. Many show robust associations with synaptotoxicity, energy metabolism, ER stress, or neuroinflammation. Validation in large cohorts is needed.	[168,170,175,179,180,181,182]
**Speculative/Exploratory**	- Caspase-8 - Various chemokines/interleukins - Other mitochondrial markers identified by deep-profiling proteomics (beyond PRDX3) - Immune system proteins with high intra-cohort variability	Biomarkers mainly identified in single studies, with limited reproducibility or associated with very general pathways (inflammation, apoptosis). Potentially relevant to pathogenesis, but clinical utility is not yet supported.	[167,175,176,177,178]

## Data Availability

No new data were created or analyzed in this study.

## Abbreviations

The following abbreviations are used in this manuscript:

α2M	Alpha2 Macroglobulin
Aβ	Amyloid beta protein
α-TNF	α-tumor necrosis factor
AD7c-NTP	Alzheimer-associated neuronal thread protein
AI	Artificial intelligence
AMPA	α-amino-3-hydroxy-5-methyl-4-isoxazolepropionic acid
ApoA-1	Apolipoprotein 1
ApoE	Apolipoprotein E
APP	Amyloid precursor protein
BBB	Blood–brain barrier
BACE1	βsecretase 1
CNS	Central nervous system
COX-2	Cyclooxygenase 2
CSF	Cerebrospinal fluid
DIA	Data-independent acquisition
ESI	Electrospray ionization
FTLD	Frontotemporal lobe dementia
GFAP	Glial fibrillary acidic protein
HDL	High-density lipoprotein
HS	Heparan sulfate
HSPGs	Heparan sulfate proteoglycans
IGFBP2/IGFBP7	Insulin-like growth factor binding protein 2/7
IL-1, IL-6, IL-10	Interleukin-1, Interleukin-6, Interleukin-10
IL-1β	Interleuki 1β
JAM-B	Junctional adhesion molecule B
LC	Liquid chromatography
LBD	Lewy body dementia
MALDI	Matrix-assisted laser desorption ionization
Man-Tf	Mannosylated glycan transferrin
MCI	Mild cognitive impairment
MIF	Macrophage Inhibitory Factor
MMP-9	Matrix metalloproteinase-9
MRI	Magnetic resonance imaging
MRM	Multiple reaction monitoring
MS	Mass spectrometry
NCAM1	Neuronal cell adhesion molecule 1
NfL	Neurofilament light
NFTs	Neurofibrillary tangles
NMDA	N-methyl-D-aspartate
NPTX2	Neuropentraxin 2
p-tau	Hyperphosphorylated tau protein
PET	Positron emission tomography
PKM	Pyruvate kinase M
PP	Pancreatic polypeptide
PRDX3	Mitochondrial thioredoxin-dependent peroxide reductase
PSEN1/PSEN2	Presenilin 1/Presenilin 2
SNAP-25	Synaptosomal-associated protein 25
(s)TREM2	(Soluble) triggering receptor of myeloid cells 2
SYT-1	Synaptotagmin-1
S100A9	S100 calcium binding protein A9
t-tau	Total tau protein
TMT	Tandem-mass-tag
TOF	Time-of-flight
YKL-40	Chitinase-3-like protein 1
YWHAG	Tyrosine 3-monooxygenase/tryptophan 5-monooxygenase activation
	protein gamma
2DE	Two-dimensional electrophoresis
